# Early (years) reactions: comparative analysis of early childhood policies and programs during the first wave of the COVID-19 pandemic

**DOI:** 10.1186/s12889-022-13344-0

**Published:** 2022-07-19

**Authors:** Joanne Kearon, Sarah Carsley, Meta van den Heuvel, Jessica Hopkins

**Affiliations:** 1 Department of Health Research Methods, Evidence, and Impact, McMaster University, Hamilton, ON Canada; 2 Public Health Ontario, Toronto, ON Canada; 3 Division of Paediatric Medicine, Hospital for Sick Children, Toronto, ON Canada; 4 Department of Pediatrics, University of Toronto, Toronto, ON Canada; 5 Dalla Lana School of Public Health, University of Toronto, Toronto, ON Canada

**Keywords:** COVID-19, Child development, Child health, Public policy

## Abstract

**Background:**

During the first wave of COVID-19 there was little evidence to guide appropriate child and family programs and policy supports.

**Methods:**

We compared policies and programs implemented to support early child health and well-being during the first wave of COVID-19 in Australia, Canada, the Netherlands, Singapore, the UK, and the USA. Program and policy themes were focused on prenatal care, well-baby visits and immunization schedules, financial supports, domestic violence and housing, childcare supports, child protective services, and food security.

**Results:**

Significant heterogeneity in implementation of OECD-recommended policy responses was found with all of the included countries implementing some of these policies, but no country implementing supports in all of the potential areas.

**Conclusions:**

This analysis gives insight into initial government reactions to support children and families, and opportunities for governments to implement further supportive programs and policies during the current pandemic and future emergencies.

**Supplementary Information:**

The online version contains supplementary material available at 10.1186/s12889-022-13344-0.

## Background

Healthy growth and development, from the prenatal environment to the first 5 years of life, plays a major role in health and well-being across the life course [1]. Social and environmental factors, such as family structure, socioeconomic status, housing, and nutrition, during early childhood influences the probability of future obesity, heart disease, mental health, educational outcomes and involvement in the criminal justice system [2]. In particular, adverse childhood experiences (ACEs), such as domestic violence, being the child of a parent with a mental health condition, or experiencing abuse or neglect, are associated with long-term stress-related changes in the nervous, endocrine and immune systems, leading to multiple poor health conditions such as type 2 diabetes, mental disorders, and cognitive decline [3].

Policies and programs that promote positive social and physically active environments for young children and their families have the potential to improve early childhood development, and subsequently health for entire populations. For example, across several countries from a range of political legacies, those that provided mothers with longer maternity leaves and higher maternity pays had higher rates of breastfeeding initiation, which promotes sensory and cognitive development, and is associated with improved infant health and behavioural outcomes [4]. Similarly, countries that publicly funded early childhood education had higher literacy rates at 15 years of age than those that were privately funded [5]. Early childhood education can also positively impact motor development, cognitive function, emotional wellbeing, and social relationships through play [6]. Early childhood food supplementation programs have been found to improve childhood nutrition and psychosocial development, particularly if those programs are delivered through childcare centres [7]. Parental income supports, including child tax credits, have also been found to prevent and mitigate the effects of ACEs and improve long-term health outcomes and educational attainment [8, 9]. Furthermore, programs that target specific populations can decrease social inequity within countries (e.g., evidence-based home visiting programs for vulnerable first-time mothers such as nurse-family partnership) [10, 11]. Thus, governmental policies and programs are able to impact early childhood health and development.

The World Health Organization declared COVID-19 a pandemic on March 11, 2020. In response to rising cases and deaths, numerous countries globally implemented community-wide public health measures such as quarantine, isolation, and closure of community services, workplaces and businesses. These very significant societal changes have had consequences on child health, parent health and family functioning [12, 13]. Quarantine and isolation indoors and loss of work can lead to significant parental stress through social isolation and loss of income. This in turn can impact children through poverty, abuse or neglect, decreased time outdoors to play, engage in physical activity and socialize, and potentially increase sedentary behaviours and screen time. Childcare and schools often serve as settings that can mitigate challenges in the home setting, such as by providing food to low-income children at risk of food insecurity, other health services (eg. vaccinations, mental health services), and holistic healthy physical and cognitive growth and development programming. In addition, COVID-19 has drastically changed how public health services, prenatal, neonatal and pediatric care are delivered. As such, many of the programs created to support early childhood development have been more difficult to access or implement during a global pandemic. Several papers have considered how health and social services can be delivered safely during the pandemic without diminishing access, quality and effectiveness of care [14–17]. Publications have highlighted gaps that already existed in supporting families in some countries [12, 18], and how the pandemic may widen inequities in prenatal and obstetric care [19]. Recommendations have been made to implement policies that can mitigate the impact of COVID-19 on children, such as reducing barriers to accessing social support, providing support for women at risk of domestic violence, and increased support for education for children that lack technology resources [20]. The first wave of the pandemic was an acute emergency and marked by incredible uncertainty for all of society. Understanding how countries responded to the needs of their families in this context may help to inform future emergency preparedness.

The objective of this paper is to compare the policies and programs that different countries put into place to support child health during the first wave of the COVID-19 pandemic. We aim to contribute to the exploration of the acute impacts of the COVID-19 pandemic on policies and programs to support early childhood development, and identify opportunities for improvement. In addition, we explore the reasons and potential impact of varying approaches on early child health and well-being with a view to improvements in emergency preparedness and response.

## Methods

To answer the question, “How did governments support child health during the COVID-19 pandemic through policies and programs?,” we compared the policies and programs implemented by governments in selected countries during their first wave of COVID-19.

### Country and government selection

The following countries were included in this study: Canada (the federal government of Canada and selected provinces of British Columbia, Alberta, Ontario and Quebec), United Kingdom (UK), Australia, Netherlands, United States of America (USA), and Singapore. In some instances, sub-national territories were included as federal responsibility for some health and social programs may be shared or devolved. Country selection was discussed and finalized amongst all authors. The focus was on the Canadian experience, with information being gathered about both federal and provincial policy interventions. The included provinces were chosen due to their comprising the majority of the Canadian population (> 85%), as well as being most affected by COVID-19 during the first wave. We then selected Organization of Economic Cooperation and Development (OECD) comparator countries, as well as Singapore, representing a variety of political legacies and COVID-19 epidemiologic experiences [21]. In the UK, Australia, Netherlands and Singapore, only federal policies were included due to the structure of their government. Within the USA, in addition to federal policies being explored, Michigan was also chosen for further comparison due to its similarities to Ontario, for which it has previously been used as a comparator to Ontario [22].

### Baseline country characteristics and COVID-19 epidemiology

Baseline information about each country was gathered in order to provide insight into the context in which decisions around policies and programs to support families are being made, including prior indicators of early childhood supports, and inform comparison between settings. These measures included population size, Gross Domestic Product (GDP) per capita, percentage (%) of GDP spent on early childhood education (ECE), enrollment of children 3–5 years in ECE, immunization coverage, and Gini coefficient. The Gini coefficient is a measure of income inequality within a group of people, on a range from 0 to 1, with higher numbers indicating greater inequality [23]. The main sources of data were the World Bank [24] and OECD [25–27], though in some cases, country specific reports were used due to lack of synthesized international data.

We also compared the countries’ experiences of the COVID-19 pandemic during their first wave by collecting data on date of first case, date of the first peak in cases, the incidence during the peak, their testing capacity during the first wave, and the Government Stringency Index (GSI) during their peak, which is a measure of the intensity of a country’s community-wide public health measures. This information was found through Our World in Data [28–30].

### Search strategy and data sources

The search strategy was designed to identify policies, programs, announcements, and guidelines released from governmental and public health organizations within each country related to children, parents, families, early childhood development, adverse childhood experiences, child welfare, pre-school, childcare or daycares. Specifically, we were interested in clinical guidelines, financial benefits or government funding of health or social programs, and childcare supports. For the purposes of this paper, a policy is any long-term change in law or regulation, while a program is a shorter-term or temporary activity that includes an active intervention. Public health organizations were included as they are often considered a branch of government, or were heavily involved in advising the government on measures related to the COVID-19 pandemic, potentially including measures to support families. Clinical guidelines were included as access to healthcare is a key determinant in early childhood development. Since the documents that were being considered are not typically peer-reviewed or published in academic literature, we focused on a search of the grey literature.

We designed and reviewed the search strategy with a health sciences librarian from Public Health Ontario. We devised search strings based on our targeted policy topics and repeated the search on a range of pre-designed search engines that pulled information specifically from governmental and public health bodies, as well as on Google. For each search string on each search engine, the first 100 results for each search were reviewed. Based on the findings of this search, targeted iterative manual searches on Google were also completed. Following this, we conducted a manual search of each country’s governmental websites (federal and provincial or state as relevant) to ensure that no other policy decisions were missed by the original search strategy. A manual search of the Netherland’s policies was completed by an author (MH) fluent in the language and then translated to English. We also reviewed guidance documents from national and provincial/state clinical colleges or bodies in pediatrics, obstetrics, and family medicine. The search was performed from August to November 2020. The full search strategy is outlined in Appendix A.

We chose to focus on early childhood development, including the prenatal environment to pre-school-aged children (0–5 years), and therefore any policies or programs specific to school-aged children were excluded (though policies that would apply to both pre-school- and school-aged children were included). Supports for families with young children were included, as the family environment is a key determinant of early childhood development. However, policies that were open to the general public, but not specifically targeting children, parents or families, were excluded. We included policies introduced by these governments from January to August 2020, as this timeframe included the first wave of COVID-19 for all countries.

During analysis, the authors discussed the policies and programs identified through the search, grouped these into the themes of prenatal and pediatric care, parental supports, and childcare and early childhood development, and then created further sub-themes based on consensus.

## Results

### Baseline characteristics of selected countries

Table 1 provides an overview of the baseline characteristics of the included countries. While each country is unique, there are similarities allowing for useful comparison. In terms of population size, the range was from Singapore’s population of 5.9 million to USA’s 328.2 million [24]. While Singapore has the smallest population, it has the highest population density. The GDP per capita ranged from $46,483 in the UK to $98,520 in Singapore, compared to $46,611 in Canada [24]. In this group of countries, the Gini coefficient ranged from 0.285 in the Netherlands to 0.458 in Singapore [27, 31].

**Table 1:** Baseline characteristics of included countries

	Population (millions)^*a*^	Population Density (/km^2^)^*b*^	GDP per capita, PPP (current international $)^*a*^	% of GDP Spent on ECE^*c*^	Enrollment of children 3–5 years old in ECE (%)^*c*^	Measles immunization coverage (%)^*d*^	Gini coefficient^*e*^	Responsibility for health and social programs and services^f^
Australia	25.4	3	53,330	0.66	84	95	0.325	Federal: funding through transfer payments to states States: delivery programs/services; additional funding above federal transfer payments
Canada	37.6	4	46,611	0.20	24	90	0.303	Federal: funding through transfer payments to provinces/territories Provincial/territorial: delivery of programs/services; additional funding above federal transfer payments
Netherlands	17.3	518	59,268	0.60	95	94	0.285	National
Singapore	5.9	8019	98,520	0.19	84	95	0.458	National
UK	66.8	278	46,483	0.65	100	91	0.366	National Devolved delivery in Scotland, Wales and Northern Ireland

^a^as of 2020 [24]; PPP-purchasing power parity rounded to nearest dollar

^b^as of 2020 [24];

^c^for ages 0-6 years old in 2006 for Canada [25], for ages 4-6 years old in 2011 for Singapore [32], and in 2015 for all other countries [26]; GDP by year was used as PPP was not available

^d^in 2019 [33];

^e^in 2016 for Singapore [31], in 2018 for all other countries [25]

^f^Refers to over-arching roles and responsibilities as there are many exceptions and nuances to health and social program/service funding and delivery. Federal levels of government generally provide limited delivery of health and social services (e.g., military). Local administrative (e.g., municipal) contributions to funding and delivery were not included

We also examined baseline information about the current status of child health and supports for children in the country which provided context for the overall health of children in the pre-pandemic period (Table 1). In some cases, there was heterogeneity in the way the data were reported between countries, and the year of the most recent data available. The amount spent on ECE, as a percentage of GDP, ranged from about 0.20% in Singapore in 2011 (4–6 years old) and Canada in 2006 (0–6 years old), to over 0.65% in the UK and Australia in 2015 (3–5 years old) [25, 26, 32]. The percentage of children 3–5 years old (0–6 years old in Canada and 4–6 years old in Singapore) enrolled in ECE ranged from 24% in Canada in 2006 to 100% in the UK in 2017 [25, 26]. Immunization coverage for all of the childhood vaccines in all countries examined was > 90%. The largest differences were seen with measles vaccine coverage, which ranged from 90% in Canada and the USA, to 95% in Australia and the UK [33].

### Epidemiologic indicators during the first wave of the COVID-19 pandemic

An overview of the epidemiology of COVID-19 in the included countries can be seen in Table 2. All countries identified their first case in late January or early February 2020, with the exception of the Netherlands, which identified their first case in late February. The date of the first wave peak ranged from late March to late July [29]. At peak, the number of cases per day ranged from 15.0 cases/million people in Australia to 203.5 cases/million people in the USA [29]. GSI scores indicate that public health measures implemented during each country’s peak were most relaxed in the USA, and the most stringent in Singapore [28]. Testing capacity and test positivity at peak was also assessed. Australia had the second highest testing capacity (2.62 per 1000), but the lowest test positivity (0.7%), while the USA had the highest testing capacity (2.89 per 1000), but mid-range test positivity (8.9%) [30]. On the other hand, the Netherlands, Singapore and the UK had lower testing capacities, and high test positivity rates (19.1–28.9%) [30]. Canada testing capacity was 0.7 per 1000 with a test positivity of 6.6% at the peak of the first wave [30].

**Table 2:** Comparison of epidemiology of COVID-19 in included countries until August 31, 2020

	Date of 1st reported case	Date of peak new cases per day	Peak new cases per day/million	GSI at date of peak new cases	Testing capacity per 1000 people at peak new cases	Test positivity at peak (%)
Australia	January 25	March 30	15.0	79.17	2.62	0.7
Canada	January 26	May 4	47.7	72.69	0.7	6.6
Netherlands	February 28	April 15	65.4	79.63	0.33	21.5
Singapore	January 24	April 27	171.8	85.19	0.54	28.9
UK	February 1	April 24	71.4	79.63	0.37	19.1
USA	January 21	July 23	203.5	67.13	2.89	8.9

^a^[28];

^b^[30];

^c^[30]; testing policies differed by country and in some cases by sub-national level. Percentage positivity should be interpreted in the context of testing eligibility (e.g., many countries limited testing to travellers to countries with known COVID-19 cases until local transmission was identified and testing capacity which was low early in the pandemic

### Healthcare: prenatal, neonatal and pediatric care

Table 3 gives an overview of the overarching recommendations and guidance from clinical professional agencies and organizations from each country related to prenatal, neonatal and pediatric care.

**Table 3:** Comparison of policies regarding prenatal and pediatric care in selected countries during COVID-19 [35–38]

Country	Jurisdiction	Prenatal Care	Well-baby visit schedule	Vaccines
Australia	Federal	• Continue routine antenatal care, though provider can arrange for extra scans if COVID-19 positive	• Continue routine schedule with mix of virtual and in-person visits	• Recommendation to continue vaccinations as scheduled
Canada	Federal	• Modified schedule, with mix of virtual and in-person practice • Consider delaying routine appointments for pregnant patients being tested or COVID-19 positive	• Modified schedule with mix of virtual and in-person visits	• Recommendation to continue vaccinations as scheduled
	Alberta	• Prenatal classes suspended	• Public health nurse or midwife to continue postpartum care as usual • Physician visits should continue, but may be virtual depending on location	• Routine immunizations to continue, with exception of school program
	British Columbia	• Reduced antenatal visits, with a mix of virtual and in-person practice	• At the discretion of physician	• Routine immunization schedule to continue
	Ontario	• Modified prenatal visit schedule	• Modified well-child schedule, with mix of in-person and virtual	• Routine infant vaccination schedule • Consider delaying 4–6 year old immunizations
	Quebec	• Modified schedule, with mix of virtual and in-person practice	• Routine schedule to be continued, though may be virtual	• Routine infant vaccination schedule
Netherlands		• Continue routine schedule	• Combination of in person and virtual visits • In-person weight checks by appointment only if there are concerns	• Routine schedule to continue
Singapore		• Postpone non-critical appointments if on a Stay-Home Notice or quarantine • Otherwise, continue routine schedule		• Continue routine schedule
UK		• Continue routine antenatal care, although can be modified, unless suspected or confirmed COVID-19	• Continue routine 6–8 week infant examination	• Continue routine childhood vaccinations as scheduled
USA	Federal	• Continue to provide medically necessary prenatal care, referrals and consultations but can modify/reduce if risk outweighs benefit	• Continue with routine well-baby visits	• Recommendation to continue infant and toddler vaccinations as scheduled
	Michigan	• Policy varied by health service provider • General reduction in in-person visits	• At the discretion of physician	• Continue routine schedule

Overall, there was general uniformity within the recommendations across countries, with only small variations. Each clinical body provided information on infection prevention and control (IPAC) policies, or links to related resources. As well, most clinical guidance focused on the clinical treatment of COVID-19 in patients (eg. pregnant women, infants). Of particular relevance was the guidance provided to health care providers on how to alter patient care schedules in order to reduce in-person contact. Most clinical organizations recommended continuing with current care schedules (ie. prenatal care, well-baby care). Canadian organizations recommended integrating virtual appointments when possible into the schedule, both for prenatal and pediatric care [14, 34]. As well, all countries’ clinical bodies recommended some flexibility in the care schedule if the patient were to have COVID-19 or be suspected to have COVID-19 - in those situations, a delay of 2 weeks would be permissible. Only Singapore recommended prenatal appointments be delayed if there was a general lockdown [35]. The USA recommended that the prenatal schedule be reduced if the risk outweighed the benefit [36]. Finally, there was agreement that the infant series of vaccination should continue as scheduled, as it was deemed an essential service in all countries.

There were a few other notable variations. For example, most jurisdictions specified that only one support person could accompany a patient during labour, while the Netherlands and Quebec allowed for two [37, 38]. As well, there was variation in the degree that different clinical organizations or agencies recommended that healthcare providers perform their own risk assessment based on their location and individual patients.

### Parental supports

Table 4 provides an overview of governmental policies and programs to support mothers and/or parents. The included policies and programs were found to be mostly related to financial supports, domestic violence and housing.

**Table 4:** Comparison of additional parental supports offered by governments in response to COVID-19 in selected countries [59–61, 63]

Country	Jurisdiction	Financial Supports	Domestic Violence and Housing	Other
Australia	Federal	• One-time payment of $750(AUD) to anyone who receives Family Tax Benefit	• $150 million (AUD) to support community organizations addressing domestic violence	
Canada	Federal	• One-time top-up of $300 (CAD) for Canada Child Benefit per child • Creation of Canada Recovery Caregiving Benefit, to provide income support for parents that must stay home to care for sick children during COVID-19	• Creation of new shelters for Indigenous women and children • Increased financial support of women’s shelters • Virtual domestic violence supports for military personnel	• Funded research on the social impacts of COVID-19 on children and families
	Alberta			
	British Columbia	• Additional $225/month (CAD) for children with special needs		
	Ontario	• One-time payment of $200–$250(CAD) per child	• Increased funding to support victims of domestic violence	
	Quebec			
Netherlands				• Funding research on the impact of COVID-19 on maternal mental health
Singapore		• One-time payment of $1000(SGD) to low-income families affected by COVID-19 • Increased child benefit by $300(SGD) for each parent in household for one month • One-time additional support for newborns, in order to encourage families to have children during COVID-19		
UK			• Introduced laws strengthening protections and increasing assistance to those experiencing domestic violence (was already underway, completed during COVID-19)	• Funding research on the impact of COVID-19 on maternal mental health
USA	Federal	• No change to federal Child Tax Credit	• CARES Act includes $% million for emergency shelter via the Family Violence Prevention and Services Act	
	Michigan			

In terms of financial supports, Canada, Australia and Singapore created specific benefits for parents, in addition to pre-existing supports, as well as financial supports that were offered to the general population during COVID-19 [39–41]. Within Canada, British Columbia provided an additional $225(CAN)/month benefit for children with special needs [42], and Ontario provided a one-time $200–$250(CAN) payment per child additional to the federal benefit [43]. Notably, there was variation in how these benefits were accessed, with Canada and Australia both requiring application to the benefits, while Singapore provided the benefit automatically to anyone who was already receiving other child benefits [39–41]. Canada introduced an additional financial support in August 2020 for parents who were forced to take time off of work in order to care for a child that must isolate [44]. Singapore also provided an additional separate benefit for low-income families, and a benefit for newborns [39]. This latter program was explicitly created to encourage people to continue having children during the COVID-19 pandemic. The UK, Netherlands and USA did not make any changes to their current policies or programs specifically related to financial support for families, although families may have benefited from other population-wide programs (e.g., universal tax credits, income programs for workers who were temporarily laid off).

Canada and Australia both increased funding to community organizations addressing domestic violence and for women’s shelters [45, 46]. Additionally, Canada’s federal government created additional shelters specifically for Indigenous women and children [45]. During the COVID-19 pandemic, the UK passed a law that increased protections and supports for those experiencing domestic violence [47]. However, that law was already being considered and drafted prior to the emergence of COVID-19.

Canada, the UK and the Netherlands specifically funded research related to the impact of COVID-19 pandemic on women and children, including the impact on mental health [48–50].

All of the countries’ governmental websites provided general information related to maternal mental health and well-being. As well, all countries’ governmental websites provided links to community organizations, programs, or other supports that already exist to aid parents and young children. Additional information including references for information in the tables is contained in Appendix B.

### Childcare and early child development

Table 5 provides an overview of all governmental supports for early childhood development, which were found to be related to childcare, child protective services and food security. It should be noted that the term childcare refers to any form of care delivered to children aged 0–5 years, which may include pre-school, nursery school, licensed or unlicensed child care, or in-home services, as the specific way childcare is delivered in each country may vary and is typically a mix of these services.

**Table 5:** Comparison of additional supports for childcare and early childhood development by governments in response to COVID-19 in selected countries

Country	Jurisdiction	Childcare	Child Protective Services	Food Security
Australia	Federal	• Offered free childcare from April to July, 2020 during COVID-19 • No official closure of childcare centres, though many closed as parents withdrew children • Financial support for childcare centres	• Transition to mixture of virtual and in-person services	• Increased funding for emergency food relief organizations
Canada	Federal	• Emergency family care during COVID for military families		• $100 million in funding for food banks and local food organizations
	Alberta	• Childcare centres were closed, except for emergency child care centres for children of essential workers	• Child intervention services not open to public, only available by phone	
	British Columbia	• Childcare centres were closed • Temporary Emergency Relief funding was provided to childcare centres to allow for them to retain staff and maintain spots for children when they reopen • Extra supports for children of essential workers, to allow for in-own-home childcare • Affordable Child Care Benefit continued, even if child was not able to attend childcare	• Transition to mixture of virtual and in-person services	• Various grants available
	Ontario	• Childcare centres were closed, except for emergency child care centres, to provide care to children of essential workers	• Transition to mixture of virtual and in-person services	
	Quebec	• Childcare centres were closed, except for emergency child care centres, to provide care to children of essential workers		
Netherlands		• Childcare centres closed, except for children of essential workers • Continued payments for child-care, even if child care centres were closed	• Mixture of virtual and in-person services	• Increased funding for food banks
Singapore		• Increased already existing universal and targeted subsidies for childcare • Lowered fee caps on childcare, in order to make high-quality childcare more affordable • Increased supports for children in pre-school with special needs • Started KIDStart Initiative, a pilot project for children from low-income families	• Children’s protective services proactively reaching out to at-risk families, including continued in-person visits	• Created a working group to assess and address food insecurity in young families during COVID-19 • Increased food vouchers for low-income families
UK		• Childcare centres were closed, except for those of essential workers • Within 2020 budget, reduced barriers to accessing tax-free childcare	• Children’s protective services were moved fully to telephone or virtual services during the peak	• If meals were provided in schools or childcare centres, they were instructed to find a way to continue providing meals to these children
USA	Federal			• Reduced barriers to accessing the Special Supplemental Nutrition Program for Women, Infants and Children • Coronavirus Food Assistance Program provides funding for food banks
	Michigan	• Childcare centres were closed, except for emergency child care centres, to provide care to children of essential workers	• Transition to mixture of virtual and in-person services	

^a^[41];

^b^(Prime Minister of Australia, 2020);

^c^(Department of Education, Skills and Employment, Government of Australia Centre, 2020);

^d^(Department for Child Protection, 2020);

^e^[46];

^f^(Canadian Armed Forces, 2020a);

^g^[45];

^h^[53];

^i^(Government of Alberta, 2020);

^j^(Ministry of Child and Family Development, Government of British Columbia, 2021);

^k^(BC Food Security Gateway, 2020);

^l^(Ministry of Health, Government of Ontario, 2020);

^m^[51];

^n^[52];

^o^[56];

^p^(Jeugdzord Nederland, 2020);

^q^(Werkgelegenheid, 2020);

^r^[59];

^s^[63];

^t^[39];

^u^[57];

^v^[47];

^w^[61];

^x^[58];

^y^[62];

^z^[55];

^aa^(Burgio, 2020)

In Canada, childcare centres were closed across the country in an attempt to reduce transmission of COVID-19, a decision made by each province. As such, there were variations across provinces in how childcare for children of essential workers was provided. Most provinces either permitted some designated childcare centres to remain open for children of essential workers or set up emergency childcare centres. Alternatively, the British Columbia government provided financial support for essential workers to arrange in-home childcare [51–54]. The Netherlands, UK and Michigan also closed childcare centres, but designated some to remain open for children of essential workers [55–57]. As childcare centres reopened, the UK introduced policy to reduce barriers to accessing tax-free childcare [58]. In contrast, Australia and Singapore kept childcare centres open throughout the first wave, although, Australia did see many childcare centres close due to parents choosing to withdraw their children [41, 59]. In response, Australia offered to pay for childcare for 2 months and also provided financial support to childcare services to help them maintain their staff until enrollment increased again [41]. Singapore introduced multiple policies to reduce barriers to high quality childcare centres, including decreasing fee caps and increasing both universal and targeted subsidies [59]. Singapore also created the KIDStart Initiative, a pilot early childhood development program for children from low-income families [59].

In terms of child protective or welfare services, almost all countries transitioned to a mix of in-person and virtual services, in order to reduce in-person contact. In Ontario, these services are managed through Ontario Children’s Aid Societies, delivered at the municipal level, creating further variation, with services continuing fully in-person in some municipalities, but not others [60]. Only England had a brief period where all services were moved to fully virtual [61]. Alternatively, Singapore child protective services began to proactively reach out to families in at-risk neighbourhoods, including in-person visits [59].

There was a large variation in the types of programs countries enacted to reduce the risk of food insecurity amongst children. Canada, the Netherlands and the USA increased funding to food banks and other emergency food relief organizations [45, 56, 62]. Three countries, the USA, Singapore and the UK, also instituted specific programs or interventions in addition to general increased funding to food relief organizations. The USA reduced barriers to accessing existing supports, such as the Special Supplemental Nutrition Program for Women, Infants and Children [62]. Singapore created a working group to assess and address food insecurity in young families, though their recommendations had not yet been made public [63]. At the same time, Singapore also increased food vouchers for low-income families [63]. The UK mandated that any childcare centres and schools that were providing food to children should find a way to continue providing that food [58].

Additionally, all of the countries’ governmental websites provided general information related to child mental health and well-being, though this very often focused more on adolescents. As well, all countries’ governmental websites provided links to community organizations, programs, or other supports that already exist to aid parents and young children.

## Discussion

### Overview

Our findings indicate that these countries provide helpful comparisons and contrasts through the variation in policy approaches in addressing maternal and early childhood concerns during the COVID-19 pandemic. Overall, there was significant heterogeneity in implementation of OECD-recommended policy responses. Overall, Singapore, Canada and Australia introduced a greater number of supports over a broader range of policy areas, while the Netherlands and USA introduced fewer policies or programs. Most policies were related to financial supports potentially indicating a gap for those who experience barriers to completing financial applications or filing taxes (e.g., undocumented residents), or where money can’t buy services (e.g., childcare if insufficient providers). It was also not clear if the financial supports were sufficient to meet needs as we did not find evidence of evaluation of the impact of implemented programs and policies. In contrast, there was relative consensus on clinical care during the prenatal, neonatal and pediatric stages.

### Reasons for variations in policies

There is likely a large range of factors that influenced the variation in policies and programs introduced by the governments of these countries. The overarching political legacy and culture of these countries may have influenced the governments’ approaches. For example, within this group of countries, the USA has a legacy of less redistributive policies and cultural attitudes that are less accepting of government intervention. Therefore, it may not be surprising that the USA also enacted fewer supports for parents and young children during the first wave of the COVID-19 pandemic [64, 65]. In comparison, Canada has a legacy of greater social welfare supports than the USA and implemented more supports for children and families during COVID-19 [65]. Interestingly, the Netherlands, which has a much larger social welfare support system than the USA and Canada, also introduced fewer policies to support parents and young children during COVID-19. Potential explanations for this observation could be a higher baseline threshold of early childhood supports and fewer restrictions in the first wave compared to other countries. For example, in the Netherlands parents are already reimbursed a percentage for child care costs, depending on their income, and all employed parents have paid sick leave and additional paid “caretaking leave” to care for a sick family member [66, 67].

### Potential impacts of policies

Preliminary studies indicated that COVID-19 and the community-wide public health measures implemented to reduce transmission of COVID-19 have already had significant impacts on maternal and child health and well-being [68]. The first wave of the COVID-19 pandemic required rapid response given the sudden disruption of long-standing systems of support, with many losing employment and access to childcare and other public health services. Furthermore, the public health measures and the closure of specific ECD programs likely disproportionately affected families already at-risk, from lower socioeconomic communities [69]. Nevertheless, the effectiveness of the policies presented here to counteract the negative impacts of COVID-19 on maternal and child health will take time to be understood.

Previous research has demonstrated the effectiveness of policies to support mothers and children in improving long-term health outcomes [11]. In line with the best available evidence, the OECD has compiled a list of possible governmental policies to counteract the negative impacts of COVID-19 on children [70]. All of the countries discussed in this study implemented some (e.g., prenatal care, child vaccination, child protection services), but not all, of the recommended actions (e.g., well child visits were discretionary in some jurisdictions, daycares closed, maternal supports for income, domestic violence and food required knowledge of available programs and in many cases application processes), suggesting significant opportunity for emergency preparedness and low barrier entry to responsive programs to protect this vulnerable group. In particular, while all governments provided information on their websites to pre-existing resources for mental health supports for young children and parents, no government created new supports or policies specifically targeting this area of need in the first wave of the COVID-19 pandemic. As well, Canada, Australia and the USA should consider how supports for early childhood development vary across their states or provinces and how this might affect health and health equity.

Moreover, it should be noted that the positive impact of these policies and programs will be mediated by the extent of implementation and uptake. For example, baseline access to large outdoor environments and having at least one parent free from working at home is a positive predictor of children’s physical activity [71]. Additionally, there is evidence that indicates that programs that automatically enroll participants and have fewer barriers to access will have greater uptake and impact than programs that require applications [72, 73]. Thus, Singapore may see a greater impact of their financial supports due to it automatically being given to those already receiving other social supports in contrast to Canada where participants had to apply for certain COVID-19 financial benefits.

### Strengths and limitations of study

To the best of our knowledge, this is the only study that has looked at the acute response of governments to support young children and families during the acute first wave period of the pandemic. This study used a comprehensive and iterative search strategy of the grey literature. This allowed us to identify all relevant policy options and programs that the intended users or recipients would have access to via public websites.

Our study is limited by the number of countries included. We purposefully chose these countries in order to provide a broad range of political traditions and COVID-19 experiences. However, since we wanted to also allow the findings to be comparable to the Canadian context, all of these countries are high-income. All of the included countries, with the exception of Singapore, are OECD comparator countries. This means that our findings may not be applicable to low- and middle- income countries. Most of the countries included share responsibilities for funding and delivery of health and social programs/services between federal, state/provincial/territorial and local administrative/municipal levels of government. Due to the large number of entities involved, it was not possible to review programs and services at all levels of government which could impact interpretation, for example if the most impactful programs were delivered at the local level. As well, though our comparison is thorough, and we briefly explored possible reasons for variations in policy choices, a much greater political analysis would be required to fully understand the reasons for each countries’ specific policy and program decisions. Lastly, while we explored each countries’ governmental response and recommendations provided by clinical colleges and bodies, the degree of uptake and implementation of these programs by the targeted population has not been explored here.

### Future directions

In the future, the implementation, uptake, and impact of these policies and programs should be assessed. Possible outcome indicators for evaluation could include infant mortality rate, low birth weight rate, vaccination coverage, educational attainment and literacy rates. Lastly, all countries should consider how to maintain a sufficient and ongoing baseline of support to optimize early childhood health and development across these policy areas, and identify in emergency planning specific program and policy areas that might require additional investment during acute and sustained emergencies.

## Conclusion

This study explored the variation in how families and young children were supported during the first wave of the COVID-19 pandemic in Canada, Australia, Netherlands, Singapore, UK and USA through governmental policies and programs, as well as changes to healthcare provision. All of the included countries implemented some policies and programs to support families and young children, but there was a large range in the number, magnitude, and accessibility of these supports. None of the countries implemented supports in all of the potential areas identified. As the COVID-19 pandemic continues, there is an opportunity for every country to provide a sufficient baseline of healthy child policies and programs to optimize early childhood outcomes, and develop comprehensive plans for future responses to acute and sustained emergencies.

## Supplementary Information

**Additional file 1:** **Appendix A.**

**Additional file 2:** **Appendix B.**

## Data Availability

All the data used in the study is from publicly available sources and data sets, which are provided in the references section.

## Abbreviations

ACE: Adverse Childhood Event
COVID-19: Coronavirus Disease 2019
ECE: Early Childhood Education
GDP: Gross Domestic Product
IPAC: Infection Prevention and Control
OECD: Organization for Economic Co-operation and Development
UK: United Kingdom
USA: United States of America

## References

[1] Maggi S, Irwin LJ, Siddiqi A, Hertzman C (2010). The social determinants of early child development: an overview. J Paediatr Child Health.

[2] Hertzman C (2010). Tackling inequality: get them while they’re young. BMJ.

[3] Danese A, McEwen BS (2012). Adverse childhood experiences, allostasis, allostatic load, and age-related disease. Physiol Behav.

[4] Andres E, Baird S, Bingenheimer JB, Markus AR (2016). Maternity leave access and health: a systematic narrative review and conceptual framework development. Matern Child Health J.

[5] van den Heuvel M, Hopkins J, Biscaro A, Srikanthan C, Feller A, Bremberg S (2013). A comparative analysis of early child health and development services and outcomes in countries with different redistributive policies. BMC Public Health.

[6] Bento G, Dias G (2017). The importance of outdoor play for young children's healthy development. Porto Biomed J.

[7] Kristjansson E, Francis DK, Liberato S, Benkhalti Jandu M, Welch V, Batal M, et al. Food supplementation for improving the physical and psychosocial health of socio-economically disadvantaged children aged three months to five years. Cochrane developmental, psychosocial and learning problems group, editor. Cochrane Database Syst Rev. 2015. 10.1002/14651858.CD009924.pub2. 10.1002/14651858.CD009924.pub2 PMC6885042 25739460

[8] Cancian M, Slack KS, Yang MY (2013). The effect of family income on risk of child maltreatment. Soc Serv Rev.

[9] Marr C, Hingtgen S, Sherman A, Windham K, Cox K. Temporarily expanding child tax credit and earned income tax credit would deliver effective stimulus, help avert poverty spike. Center on Budget and Policy Priorities 2020. https://www.cbpp.org/research/federal-tax/temporarily-expanding-child-tax-credit-and-earned-income-tax-credit-would. Accessed 13 Jan 2021.

[10] Navarro V, Muntaner C, Borrell C, Benach J, Quiroga Á, Rodríguez-Sanz M (2006). Politics and health outcomes. Lancet.

[11] Siddiqi A, Irwin LJ, Hertzman C (2011). Early child development: a powerful equalizer. Improving equity in health by addressing social determinants.

[12] Thapa SB, Mainali A, Schwank SE, Acharya G (2020). Maternal mental health in the time of the COVID-19 pandemic. Acta Obstet Gynecol Scand.

[13] Van Lancker W, Parolin Z (2020). COVID-19, school closures, and child poverty: a social crisis in the making. Lancet Public Health.

[14] Bogler T, Bogler O. Interim schedule for pregnant women and children during the COVID-19 pandemic. Can Fam Physician. 2020; https://www.cfp.ca/news/2020/03/25/3-24. Accessed 26 Jan 2021. PMC7219820 32404468

[15] Graham WJ, Afolabi B, Benova L, Campbell OMR, Filippi V, Nakimuli A (2020). Protecting hard-won gains for mothers and newborns in low-income and middle-income countries in the face of COVID-19: call for a service safety net. BMJ Glob Health.

[16] Jago CA, Singh SS, Moretti F (2020). Coronavirus disease 2019 (COVID-19) and pregnancy: combating isolation to improve outcomes. Obstet Anesth Dig.

[17] Zhang X-B, Gui Y-H, Xu X, Zhu D-Q, Zhai Y-H, Ge X-L (2020). Response to children’s physical and mental needs during the COVID-19 outbreak. World J Pediatr.

[18] Hynan MT (2020). Covid-19 and the need for perinatal mental health professionals: now more than ever before. J Perinatol.

[19] Onwuzurike C, Meadows AR, Nour NM (2020). Examining inequities associated with changes in obstetric and gynecologic care delivery during the coronavirus disease 2019 (COVID-19) pandemic. Obstet Gynecol.

[20] Douglas M, Katikireddi SV, Taulbut M, McKee M, McCartney G (2020). Mitigating the wider health effects of covid-19 pandemic response. BMJ..

[21] Canadian Institute for Health Information. OECD interactive tool: international comparisons — peer countries, Canada 2018. https://www.cihi.ca/en/oecd-interactive-tool-peer-countries-can. Accessed 20 Jan 2021.

[22] Murphy RP, Emes J, Eisen B. Ontario vs Michigan: lessons from the wolverine state. Fraser Institute. 2016; https://www.fraserinstitute.org/sites/default/files/study-ont-vs-mich.pdf. Accessed 20 Oct 2021.

[23] De Maio FG (2007). Income inequality measures. J Epidemiol Amp Community Health.

[24] World Bank. World Bank open data. In: Open Data. 2022; https://data.worldbank.org/. Accessed 27 Mar 2022.

[25] Organisation for Economic Coperation and Development. Starting strong II: early childhood education and care. In: OECD 2006. https://www.oecd-ilibrary.org/education/starting-strong-ii_9789264035461-en. Accessed 18 Jan 2021.

[26] Organisation for Economic Cooperation and Development. OECD family database - OECD [Internet]. In: OECD Family Database. 2019. https://www.oecd.org/els/family/database.htm. Accessed 18 Jan 2021.

[27] Organisation for Economic Cooperation and Development. Income distribution database. In: income distribution database. 2020. https://stats.oecd.org/Index.aspx?DataSetCode=IDD. Accessed 18 Jan 2021.

[28] Ritchie H, Ortiz-Ospina E, Beltekian D, Mathieu E, Hasell J, Macdonald B, et al. Policy responses to the coronavirus pandemic - statistics and research. In: Our World in Data. 2021; https://ourworldindata.org/policy-responses-covid. Accessed 18 Jan 2021.

[29] Ritchie H, Ortiz-Ospina E, Beltekian D, Mathieu E, Hasell J, Macdonald B, et al. Coronavirus (COVID-19) cases - statistics and research. In: Our World in Data. 2021. Available from: https://ourworldindata.org/covid-cases. Accessed 18 Jan 2021.

[30] Ritchie H, Ortiz-Ospina E, Beltekian D, Mathieu E, Hasell J, Macdonald B, et al. Coronavirus (COVID-19) testing - statistics and research. In: Our World in Data. 2021; https://ourworldindata.org/coronavirus-testing. Accessed 18 Jan 2021.

[31] Li TW (2020). Income inequality in Singapore falls to lowest level in almost two decades as household incomes rise.

[32] Early Childhood Development Agency (2012). Ensuring quality early childhood education and childcare services.

[33] Vanderslott S, Dadonaite B, Roser M (2013). Vaccination.

[34] Elwood C, Raeside A, Boucoiran I, Van Schalkwyk J, Money D, Yudin M (2020). Updated SOGC committee opinion - COVID-19 in pregnancy [internet].

[35] College of Obstetricians & Gynaecologists, Singapore. Committee opinion - management of pregnancy and birth in women with coronavirus disease (COVID-19). 2020. https://www.ams.edu.sg/view-pdf.aspx?file=media%5c5443_fi_921.pdf&ofile=(Committee+Opinion)+Management+of+Pregnancy+and+Birth+in+Women+with+Covid-19+April+(20200420).pdf. Accessed 20 Oct 2021.

[36] American College of Obstetricians and Gynecologists. COVID-19 FAQs for obstetrician-gynecologists, obstetrics. 2020. https://www.acog.org/en/ClinicalInformation/Physician FAQs/COVID 19 FAQs for Ob Gyns Obstetrics. Accessed 31 Aug 2020.

[37] Government of Quebec. Pregnancy, delivery and the postnatal period during the coronavirus disease (COVID-19) pandemic. 2020. https://www.quebec.ca/en/health/health-issues/a-z/2019-coronavirus/information-for-pregnant-women-coronavirus-covid-19/. Accessed 30 Aug 2020.

[38] Nederlandese Vereniging Voor Obstetrie & Gynaecologie. Perinatologische zorg in tijden van COVID-19. NVOG. 2020. https://www.nvog.nl/actueel/perinatologische-zorg-in-tijden-van-covid-19/. Accessed 30 Aug 2020.

[39] Government of Singapore. Singapore budget 2020 - supplementary budget statement. 2020. https://www.singaporebudget.gov.sg/budget_2020/resilience-budget/supplementary-budget-statement. Accessed 13 Jan 2021.

[40] Canada revenue agency. Canada child benefit (CCB) payment increase: CRA and COVID-19 [Internet] 2020. https://www.canada.ca/en/revenue-agency/campaigns/covid-19-update/covid-19-ccb-payment-increase.html. Accessed 13 Jan 2021.

[41] Australian Bureau of Statistics. Methods changes during the COVID-19 period. 2020. https://www.abs.gov.au/articles/methods-changes-during-covid-19-period. Accessed 30 Aug 2020.

[42] Columbia B. Ministry of Children and Family Development. Province provides emergency fund for children with special needs. 2020; https://news.gov.bc.ca/releases/2020CFD0043-000650.

[43] Ontario Ministry of Education. Archived - get support for families. 2020. https://www.ontario.ca/page/get-support-families. Accessed 13 Jan 2021.

[44] Canada Revenue Agency Canada Recovery Caregiving Benefit (CRCB) 2020. https://www.canada.ca/en/revenue-agency/services/benefits/recovery-caregiving-benefit.html. Accessed 24 Jan 2021.

[45] Department of Finance Canada. Canada’s COVID-19 economic response plan. 2020. https://www.canada.ca/en/department-finance/economic-response-plan.html. Accessed 13 Jan 2021.

[46] Murphy K. Australian government pumps $1bn into health and family violence services as coronavirus spreads. In: The Guardian. 2020; http://www.theguardian.com/australia-news/2020/mar/29/australian-government-to-pump-1bn-into-health-and-family-violence-services-as-coronavirus-spreads. Accessed 13 Jan 2021.

[47] UK Government. Domestic abuse bill 2020: overarching factsheet. 2020. https://www.gov.uk/government/publications/domestic-abuse-bill-2020-factsheets/domestic-abuse-bill-2020-overarching-factsheet. Accessed 13 Jan 2021.

[48] Canadian Institutes of Health Research. Government of Canada funds 49 additional COVID-19 research projects – details of the funded projects. 2020. https://www.canada.ca/en/institutes-health-research/news/2020/03/government-of-canada-funds-49-additional-covid-19-research-projects-details-of-the-funded-projects.html. Accessed 13 Jan 2021.

[49] Dutch research council (NWO). Corona: fast-track data. 2020. https://www.nwo.nl/en/researchprogrammes/corona-fast-track-data. Accessed 13 Jan 2021.

[50] Economic and Social Research Council. Social science and COVID-19 2020. https://esrc.ukri.org/files/news-events-and-publications/news/esrc-covid-19-activity/. Accessed 30 Aug 2020.

[51] Government of Ontario. Ontario offers emergency child care to more frontline staff. 2020. https://news.ontario.ca/en/release/56696/ontario-offers-emergency-child-care-to-more-frontline-staff. Accessed 30 Aug 2020.

[52] Government of Quebec. Questions and answers on education and families during the COVID-19 pandemic. 2020. https://www.quebec.ca/en/health/health-issues/a-z/2019-coronavirus/answers-questions-coronavirus-covid19/questions-answers-education-families-covid-19/. Accessed 30 Aug 2020.

[53] Johnson L (2020). COVID-19: Alberta expands eligibility for child care to include all essential workers.

[54] British Columbia Ministry of Child and Family Development. Ministry of Children & Family Development response to COVID-19. 2020. https://www2.gov.bc.ca/gov/content/family-social-supports/covid-19-information. Accessed 13 Jan 2021.

[55] Government of Michigan. Whitmer - executive order 2020–51: expanding child care access during the COVID-19 pandemic - RESCINDED. 2020. https://www.michigan.gov/whitmer/0,9309,7-387-90499_90705-526011%2D%2D,00.html. Accessed 30 Aug 2020.

[56] Government of Netherlands. Childcare for children of people working in crucial sectors 2020. https://www.government.nl/topics/coronavirus-covid-19/documents/publications/2020/03/20/childcare-for-children-of-people-working-in-crucial-sectors. Accessed 30 Aug 2020.

[57] Key worker: official list of UK personnel who can still send children to school. In: The Guardian 2020. http://www.theguardian.com/world/2020/mar/20/key-worker-official-list-of-uk-personnel-who-can-still-send-children-to-school. Accessed 30 Aug 2020.

[58] Working Families. Coronavirus (COVID-19) – what financial support is there for working families?. 2020. https://workingfamilies.org.uk/articles/coronavirus-support/. .

[59] Channel news Asia. MSF to strengthen social safety nets ensuring ‘no Singaporean is left behind’ amid COVID-19: Masagos Zulkifli 2020. https://www.channelnewsasia.com/news/singapore/covid-19-msf-strengthen-social-safety-nets-masagos-zulkifli-13051466. Accessed 13 Jan 2021.

[60] Ontario Ministry of Child and Youth Services. About Ontario’s children aid societies. 2020. http://www.children.gov.on.ca/htdocs/English/professionals/childwelfare/societies/index.aspx. Accessed 30 Aug 2020.

[61] Government of United Kingdom. Coronavirus (COVID-19): guidance for children’s social care services. 2020. https://www.gov.uk/government/publications/coronavirus-covid-19-guidance-for-childrens-social-care-services/coronavirus-covid-19-guidance-for-local-authorities-on-childrens-social-care. Accessed 30 Aug 2020.

[62] Food and Nutrition Service, U. S department of agriculture. FNS Responds to COVID-19 2020. https://www.fns.usda.gov/coronavirus. Accessed 30 Aug 2020.

[63] Singapore Ministry of Social and Family Development. Impact of COVID-19 on Singaporeans and supporting measures. 2020. https://www.msf.gov.sg/media-room/Pages/Impact-of-COVID-19-on-Singaporeans-and-Supporting-Measures.aspx. Accessed 13 Jan 2021.

[64] Fishback P. Social welfare expenditures in the United States and the Nordic countries: 1900-2003. In: Cambridge, MA: National Bureau of Economic Research; 2010. http://www.nber.org/papers/w15982.pdf. .

[65] Stefan Svallfors. Welfare regimes and welfare opinions: a comparison of eight Western countries. In: Vogel J, editor. European welfare production: institutional configuration and distributional outcome. Dordrecht: Springer Netherlands; 2003. p. 171–196.

[66] Pfau-Effinger B (2005). Welfare state policies and the development of care arrangements. Eur Soc.

[67] Yerkes MA, Javornik J (2019). Creating capabilities: childcare policies in comparative perspective. J Eur Soc Policy.

[68] Ontario Agency for Health Protection and Promotion (public health Ontario) Negative impacts of community-based public health measures on children, adolescents and families during the COVID-19 pandemic: update. 2021. https://www.publichealthontario.ca/-/media/documents/ncov/he/2021/01/rapid-review-neg-impacts-children-youth-families.pdf?la=en. Accessed 20 Oct 2021.

[69] Spinelli M, Lionetti F, Setti A, Fasolo M (2020). Parenting stress during the COVID-19 outbreak: socioeconomic and environmental risk factors and implications for children emotion regulation. Fam Process.

[70] Organisation for Economic Co-operation and Development. Combatting COVID-19’s effect on children. 2020. http://www.oecd.org/coronavirus/policy-responses/combatting-covid-19-s-effect-on-children-2e1f3b2f/. Accessed 15 Sep 2020.

[71] Pombo A, Luz C, Rodrigues LP, Ferreira C, Cordovil R (2020). Correlates of children's physical activity during the COVID-19 confinement in Portugal. Public Health.

[72] Dorn S (2007). Automatic enrollment strategies: helping state coverage expansions achieve their goals.

[73] Hefford M, Crampton P, Foley J (2005). Reducing health disparities through primary care reform: the New Zealand experiment. Health Policy.

