# Supplementary material for: Early (years) reactions: comparative analysis of early childhood policies and programs during the first wave of the COVID-19 pandemic
Source: BMC Public Health. 2022 Jul 19;22:1383. doi: 10.1186/s12889-022-13344-0 (PMC9295085; doi:10.1186/s12889-022-13344-0)
Supplement: Supplementary file 2 — Additional file 2: Appendix B. [file 12889_2022_13344_MOESM2_ESM.docx]

**Appendix B: Tables with citations**

Table 1. Baseline characteristics of included countries

|  | Population (millions)*^a^* | Population Density (/km^2^)*^b^* | GDP per capita, PPP (current international $)*^a^* | % of GDP Spent on ECE*^c^* | Enrollment of children 3-5 years old in ECE (%)*^c^* | Measles immunization coverage (%)*^d^* | Gini coefficient*^e^* | Responsibility for health and social programs and services^f^ |
| --- | --- | --- | --- | --- | --- | --- | --- | --- |
| Australia | 25.4 | 3 | 53 330 | 0.66 | 84 | 95 | 0.325 | Federal: funding through transfer payments to states  States: delivery programs/services; additional funding above federal transfer payments |
| Canada | 37.6 | 4 | 46 611 | 0.20 | 24 | 90 | 0.303 | Federal: funding through transfer payments to provinces/territories  Provincial/territorial: delivery of programs/services; additional funding above federal transfer payments |
| Netherlands | 17.3 | 518 | 59 268 | 0.60 | 95 | 94 | 0.285 | National |
| Singapore | 5.9 | 8019 | 98 520 | 0.19 | 84 | 95 | 0.458 | National |
| UK | 66.8 | 278 | 46 483 | 0.65 | 100 | 91 | 0.366 | National  Devolved delivery in Scotland, Wales and Northern Ireland |
| USA | 328.2 | 36 | 63 593 | 0.33 | 66.1 | 90 | 0.390 | Federal: funding through transfer payments to states  States: delivery of programs/services; additional funding above federal transfer payments |

*^a^* as of 2020 (World Bank, 2022); PPP-purchasing power parity rounded to nearest dollar

*^b^* as of 2020 (World Bank, 2022);

*^c^* for ages 0-6 years old in 2006 for Canada (OECD, 2006), for ages 4-6 years old in 2011 for Singapore (Early Childhood Development Agency, 2012), and in 2015 for all other countries (OECD, 2019); GDP by year was used as PPP was not available.

*^d^* in 2019 (Vanderslott et al., 2013);

*^e^* in 2016 for Singapore (Li, 2020), in 2018 for all other countries (OECD, 2020)

^f^ Refers to over-arching roles and responsibilities as there are many exceptions and nuances to health and social program/service funding and delivery. Federal levels of government generally provide limited delivery of health and social services (e.g., military). Local administrative (e.g., municipal) contributions to funding and delivery were not included.

Table 2. Comparison of epidemiology of COVID-19 in included countries until August 31, 2020

|  | Date of 1^st^ reported case*^a^* | Date of peak new cases per day*^a^* | Peak new cases per day/million*^a^* | GSI at date of peak new cases*^b^* | Testing capacity per 1000 people at peak new cases*^c^* | Test positivity at peak (%)*^c^* |
| --- | --- | --- | --- | --- | --- | --- |
| Australia | January 25 | March 30 | 15.0 | 79.17 | 2.62 | 0.7 |
| Canada | January 26 | May 4 | 47.7 | 72.69 | 0.7 | 6.6 |
| Netherlands | February 28 | April 15 | 65.4 | 79.63 | 0.33 | 21.5 |
| Singapore | January 24 | April 27 | 171.8 | 85.19 | 0.54 | 28.9 |
| UK | February 1 | April 24 | 71.4 | 79.63 | 0.37 | 19.1 |
| USA | January 21 | July 23 | 203.5 | 67.13 | 2.89 | 8.9 |

*^a^* (Ritchie et al., 2021a);

^b^ (Ritchie et al., 2021c);

*^c^* (Ritchie et al., 2021c); testing policies differed by country and in some cases by sub-national level. Percentage positivity should be interpreted in the context of testing eligibility (e.g., many countries limited testing to travellers to countries with known COVID-19 cases until local transmission was identified and testing capacity which was low early in the pandemic.

Table 3. Comparison of policies regarding prenatal and pediatric care in selected countries during COVID-19

| Country | Jurisdiction | Prenatal Care | Well-baby visit schedule | Vaccines |
| --- | --- | --- | --- | --- |
| Australia | Federal | - Continue routine antenatal care, though provider can arrange for extra scans if COVID-19 positive^a^ | - Continue routine schedule with mix of virtual and in-person visits^b^ | - Recommendation to continue vaccinations as scheduled^c^ |
| Canada | Federal | - Modified schedule, with mix of virtual and in-person practice^d^ - Consider delaying routine appointments for pregnant patients being tested or COVID-19 positive^e^ | - Modified schedule with mix of virtual and in-person visits^d^ | - Recommendation to continue vaccinations as scheduled^f^ |
|  | Alberta | - Prenatal classes suspended^g^ | - Public health nurse or midwife to continue postpartum care as usual^g^ - Physician visits should continue, but may be virtual depending on location^g^ | - Routine immunizations to continue, with exception of school program^h^ |
|  | British Columbia | - Reduced antenatal visits, with a mix of virtual and in-person practice^i^ | - At the discretion of physician^j^ | - Routine immunization schedule to continue^k^ |
|  | Ontario | - Modified prenatal visit schedule^d^ | - Modified well-child schedule, with mix of in-person and virtual^d^ | - Routine infant vaccination schedule^d^ - Consider delaying 4-6 year old immunizations^d^ |
|  | Quebec | - Modified schedule, with mix of virtual and in-person practice^rl^ | - Routine schedule to be continued, though may be virtual^l^ | - Routine infant vaccination schedule^m^ |
| Netherlands | | - Continue routine schedule^n^ | - Combination of in person and virtual visits^o^ - In-person weight checks by appointment only if there are concerns^o^ | - Routine schedule to continue^o^ |
| Singapore | | - Postpone non-critical appointments if on a Stay-Home Notice or quarantine^p^ - Otherwise, continue routine schedule^p^ |  | - Continue routine schedule^q^ |
| UK | | - Continue routine antenatal care, although can be modified, unless suspected or confirmed COVID-19^r^ | - Continue routine 6-8 week infant examination^s^ | - Continue routine childhood vaccinations as scheduled^s^ |
| USA | Federal | - Continue to provide medically necessary prenatal care, referrals and consultations but can modify/reduce if risk outweighs benefit^t^ | - Continue with routine well-baby visits^u^ | - Recommendation to continue infant and toddler vaccinations as scheduled^u^ |
|  | Michigan | - Policy varied by health service provider^v^ - General reduction in in-person visits^v^ | - At the discretion of physician^w^ | - Continue routine schedule^x^ |

^a^(The Royal Australian and New Zealand College of Obstetricians and Gynaecologists, 2020); ^b^(NSW Health, 2020b); ^c^(NSW Health, 2020a); ^d^(Bogler & Bogler, 2020); ^e^(Audibert et al., 2020); ^f^(Canadian Paediatric Society, 2020); ^g^(Alberta Health Services, 2020a);^h^(Alberta Health Services, 2020b); ^i^(Provincial Health Services Authority, 2020); ^j^(Health Link BC, 2020b); ^k^(Health Link BC, 2020a); ^l^(Government of Quebec, 2020a); ^m^(Government of Quebec, 2020b); ^n^(Nederlandese Vereniging Voor Obstetrie & Gynaecologie, 2020); ^o^(Jong JGZ, 2020); ^p^(College of Obstetricians & Gynaecologists, Singapore, 2020); ^q^(Health Promotion Board, Government of Singapore, 2020); ^r^(Royal College of Obstetricians & Gynaecologists, 2020); ^s^(Santhanam, 2020); ^t^(American College of Obstetricians and Gynecologists, 2020); ^u^(American Academy of Pediatrics, 2021); ^v^(Government of Michigan, 2020a); ^w^(Government of Michigan, 2020b); ^x^(Michigan Department of Health and Human Services, 2020)

Table 4. Comparison of additional maternal supports offered by governments in response to COVID-19 in selected countries

| Country | Jurisdiction | Financial Supports | Domestic Violence and Housing | Other |
| --- | --- | --- | --- | --- |
| Australia | Federal | - One-time payment of $750(AUD) to anyone who receives Family Tax Benefit^a^ | - $150 million (AUD) to support community organizations addressing domestic violence^b^ |  |
| Canada | Federal | - One-time top-up of $300 (CAD) for Canada Child Benefit per child^c^ - Creation of Canada Recovery Caregiving Benefit, to provide income support for parents that must stay home to care for sick children during COVID-19^d^ | - Creation of new shelters for Indigenous women and children^e^ - Increased financial support of women’s shelters^e^ - Virtual domestic violence supports for military personnel^f^ | - Funded research on the social impacts of COVID-19 on children and families^g^ |
|  | Alberta |  |  |  |
|  | British Columbia | - Additional $225/month (CAD) for children with special needs^h^ |  |  |
|  | Ontario | - One-time payment of $200-$250(CAD) per child^i^ | - Increased funding to support victims of domestic violence^j^ |  |
|  | Quebec |  |  |  |
| Netherlands | |  | - Country wide media campaign with information about domestic violence^k^ | - Funding research on the impact of COVID-19 on maternal mental health^l^ |
| Singapore | | - One-time payment of $1000(SGD) to low-income families affected by COVID-19^m^ - Increased child benefit by $300(SGD) for each parent in household for one month^n^ - One-time additional support for newborns, in order to encourage families to have children during COVID-19^o^ |  |  |
| UK | |  | - Introduced laws strengthening protections and increasing assistance to those experiencing domestic violence (was already underway, completed during COVID-19)^p^ | - Funding research on the impact of COVID-19 on maternal mental health^q^ |
| USA | Federal | - No change to federal Child Tax Credit^r^ | - CARES Act includes $% million for emergency shelter via the Family Violence Prevention and Services Act^s^ |  |
|  | Michigan |  |  |  |

^a^(Department of Social Services, Australian Government, 2020); ^b^(Murphy, 2020); ^c^(Canada Revenue Agency, Government of Canada, 2020a); ^d^(Canada Revenue Agency, Government of Canada, 2020b); ^e^(Department of Finance Canada, Government of Canada, 2020); ^f^(Canadian Armed Forces, 2020b); ^g^(Canadian Institutes of Health Research, Government of Canada, 2020); ^h^(Ministry of Children and Family Development, Government of British Columbia, 2020); ^i^(Ministry of Education, Government of Ontario, 2020); ^j^(Government of Ontario, 2020a); ^k^(Government of Netherlands, 2020); ^l^(Dutch Research Council (NWO), 2020); ^m^(Ministry of Social and Family Development, Government of Singapore, 2020b); ^n^(Medina, 2020); ^o^(Budget 2020, Government of Singapore, 2020b); ^p^(Home Office, UK Government, 2020); ^q^(Staniscuaski et al., 2020); ^r^(Marr et al., 2020); ^s^(National Network to End Domestive Violence, 2020)

Table 5. Comparison of additional supports for childcare and early childhood development by governments in response to COVID-19 in selected countries

| Country | Jurisdiction | Daycares and Childcare | Child Protective Services | Food Security |
| --- | --- | --- | --- | --- |
| Australia | Federal | - Offered free childcare from April to July, 2020 during COVID-19^a^ - No official closure of daycares, though many closed as parents withdrew children^b^ - Financial support for childcare centres^b,c^ | - Transition to mixture of virtual and in-person services^d^ | - Increased funding for emergency food relief organizations^e^ |
| Canada | Federal | - Emergency family care during COVID for military families^f^ |  | - $100 million in funding for food banks and local food organizations^g^ |
|  | Alberta | - Daycares were closed, except for emergency child care centres for children of essential workers^h^ | - Child intervention services not open to public, only available by phone^i^ |  |
|  | British Columbia | - Daycares were closed^j^ - Temporary Emergency Relief funding was provided to daycare centres to allow for them to retain staff and maintain spots for children when they reopen^j^ - Extra supports for children of essential workers, to allow for in-own-home childcare^j^ - Affordable Child Care Benefit continued, even if child was not able to attend daycare^j^ | - Transition to mixture of virtual and in-person services^j^ | - Various grants available^k^ |
|  | Ontario | - Daycares were closed, except for emergency child care centres, to provide care to children of essential workers^l^ | - Transition to mixture of virtual and in-person services^m^ |  |
|  | Quebec | - Daycares were closed, except for emergency child care centres, to provide care to children of essential workers^n^ |  |  |
| Netherlands | | - Daycares closed, except for children of essential workers^o^ - Continued payments for child-care, even if child care centres were closed^o^ | - Mixture of virtual and in-person services^p^ | - Increased funding for food banks^q^ |
| Singapore | | - Increased already existing universal and targeted subsidies for childcare^r^ - Lowered fee caps on childcare, in order to make high-quality childcare more affordable^r^ - Increased supports for children in pre-school with special needs^r^ - Started KIDStart Initiative, a pilot project for children from low-income families^r^ | - Children’s protective services proactively reaching out to at-risk families, including continued in-person visits^r^ | - Created a working group to assess and address food insecurity in young families during COVID-19^s^ - Increased food vouchers for low-income families^t^ |
| UK | | - Daycares were closed, except for those of essential workers^u^ - Within 2020 budget, reduced barriers to accessing tax-free childcare^v^ | - Children’s protective services were moved fully to telephone or virtual services during the peak^w^ | - If meals were provided in schools or daycares, they were instructed to find a way to continue providing meals to these children^x^ |
| USA | Federal |  |  | - Reduced barriers to accessing the Special Supplemental Nutrition Program for Women, Infants and Children^y^ - Coronavirus Food Assistance Program provides funding for food banks^y^ |
|  | Michigan | - Daycares were closed, except for emergency child care centres, to provide care to children of essential workers^z^ | - Transition to mixture of virtual and in-person services^aa^ |  |

^a^(Australian Bureau of Statistics, Australian Governme`nt, 2020); ^b^(Prime Minister of Australia, 2020); ^c^(Department of Education, Skills and Employment, Government of Australia Centre, 2020); ^d^(Department for Child Protection, 2020); ^e^(Murphy, 2020); ^f^(Canadian Armed Forces, 2020a); ^g^(Department of Finance Canada, Government of Canada, 2020); ^h^(Lisa Johnson, 2020); ^i^(Government of Alberta, 2020); ^j^(Ministry of Child and Family Development, Government of British Columbia, 2021); ^k^(BC Food Security Gateway, 2020); ^l^ (Ministry of Health, Government of Ontario, 2020); ^m^(Government of Ontario, 2020b); ^n^(Government of Quebec, 2020c); ^o^(Ministry of Health, Welfare and Sport, Government of the Netherlands, 2020); ^p^(Jeugdzord Nederland, 2020); ^q^(Werkgelegenheid, 2020); ^r^(Channel News Asia, 2020); ^s^(Ministry of Social and Family Development, Government of Singapore, 2020a); ^t^(Budget 2020, Government of Singapore, 2020a); ^u^(“Key Worker,” 2020); ^v^(HM Treasury, UK Government, 2020); ^w^(Government of United Kingdom, 2020); ^x^(Working Families, 2020); ^y^(Food and Nutrition Service, U.S Department of Agriculture, 2020); ^z^(The Office of Governor Gretchen Whitmer, Government of Michigan, 2020); ^aa^(Burgio, 2020)

**References:**

Alberta Health Services. (2020a). *COVID-19 and Pregnancy, Birth, Postpartum, and Breastfeeding: Information for Expectant and New Parents*. 10.

Alberta Health Services. (2020b, May). *Immunizations during the COVID-19 pandemic*. AHS Immunization. https://immunizealberta.ca/i-want-immunize/immunizations-during-covid-19-pandemic

American Academy of Pediatrics. (2021, January 6). *Guidance on Providing Pediatric Well-Care During COVID-19*. http://services.aap.org/en/pages/2019-novel-coronavirus-covid-19-infections/clinical-guidance/guidance-on-providing-pediatric-well-care-during-covid-19/

American College of Obstetricians and Gynecologists. (2020). *COVID-19 FAQs for Obstetrician-Gynecologists, Obstetrics*. https://www.acog.org/en/Clinical Information/Physician FAQs/COVID 19 FAQs for Ob Gyns Obstetrics

Audibert, F., Ouellet, A., Okun, N., & Wilson, R. D. (2020). *Prenatal Screening Update during the COVID-19 Pandemic*. Society of Obstetricians and Gynaecologists of Canada. https://www.sogc.org/common/Uploaded%20files/Prenatal%20Screening%20Update%20during%20the%20COVID_Final%20May13%20(2).pdf

Australian Bureau of Statistics, Australian Government. (2020, October 7). *Methods changes during the COVID-19 period*. https://www.abs.gov.au/articles/methods-changes-during-covid-19-period

BC Food Security Gateway. (2020). *Funding Opportunities Archive*. BC Food Security Gateway. https://bcfoodsecuritygateway.ca/funding/

Bogler, T., & Bogler, O. (2020). Interim schedule for pregnant women and children during the COVID-19 pandemic. *Canadian Family Physician*. https://www.cfp.ca/news/2020/03/25/3-24

Budget 2020, Government of Singapore. (2020a, July 21). *Singapore Budget 2020 | Supplementary Budget Statement*. https://www.singaporebudget.gov.sg/budget_2020/resilience-budget/supplementary-budget-statement

Budget 2020, Government of Singapore. (2020b, October 5). *Singapore Budget 2020 | Ministerial Statement Oct 2020*. https://www.singaporebudget.gov.sg/budget_2020/ministerial-statement-oct-2020/ministerial-statement-oct-2020#t2

Burgio, A. (2020, April 1). *Child Protective Services still protecting kids despite COVID-19 crisis*. WEYI. https://nbc25news.com/news/coronavirus/child-protective-services-still-protecting-kids-despite-covid-19-crisis

Canada Revenue Agency, Government of Canada. (2020a, September 29). *Canada child benefit (CCB) payment increase: CRA and COVID-19*. Aem. https://www.canada.ca/en/revenue-agency/campaigns/covid-19-update/covid-19-ccb-payment-increase.html

Canada Revenue Agency, Government of Canada. (2020b, October 2). *Canada Recovery Caregiving Benefit (CRCB)*. Aem. https://www.canada.ca/en/revenue-agency/services/benefits/recovery-caregiving-benefit.html

Canadian Armed Forces. (2020a). *Emergency Family Care*. https://www.cafconnection.ca/National/Programs-Services/For-Parents-and-Caregivers/Child-Care/Emergency-Family-Care.aspx

Canadian Armed Forces. (2020b). *Family Violence and COVID-19*. https://www.cafconnection.ca/National-Capital-Region/Adults/Health/Family-Violence-and-COVID-19.aspx

Canadian Institutes of Health Research, Government of Canada. (2020, March 19). *Government of Canada funds 49 additional COVID-19 research projects – Details of the funded projects* [Backgrounders]. Gcnws. https://www.canada.ca/en/institutes-health-research/news/2020/03/government-of-canada-funds-49-additional-covid-19-research-projects-details-of-the-funded-projects.html

Canadian Paediatric Society. (2020, April 30). *Stick to immunization schedule during the COVID-19 pandemic, paediatricians urge*. https://www.cps.ca/en/media/stick-to-immunization-schedule-during-the-covid-19-pandemic

Channel News Asia. (2020, August 25). *MSF to strengthen social safety nets ensuring ‘no Singaporean is left behind’ amid COVID-19: Masagos Zulkifli*. CNA. https://www.channelnewsasia.com/news/singapore/covid-19-msf-strengthen-social-safety-nets-masagos-zulkifli-13051466

College of Obstetricians & Gynaecologists, Singapore. (2020). *Committee Opinion—Management of Pregnancy and Birth in Women with Coronavirus Disease (COVID-19)*. https://www.ams.edu.sg/view-pdf.aspx?file=media%5c5443_fi_921.pdf&ofile=(Committee+Opinion)+Management+of+Pregnancy+and+Birth+in+Women+with+Covid-19+April+(20200420).pdf

Department for Child Protection. (2020). *Coronavirus (COVID-19)* (South Australia) [Text]. Department for Child Protection. https://www.childprotection.sa.gov.au/service-providers/coronavirus-covid-19

Department of Education, Skills and Employment, Government of Australia Centre. (2020, March 13). *Minimising the impact of COVID-19 on child care*. Ministers’ Media Centre. https://ministers.dese.gov.au/tehan/minimising-impact-covid-19-child-care

Department of Finance Canada, Government of Canada. (2020, September 14). *Canada’s COVID-19 Economic Response Plan* [Financial material]. Aem. https://www.canada.ca/en/department-finance/economic-response-plan.html

Department of Social Services, Australian Government. (2020, December 22). *Coronavirus (COVID-19) information and support*. https://www.dss.gov.au/about-the-department/coronavirus-covid-19-information-and-support

Dutch Research Council (NWO). (2020). *Corona: Fast-track data | NWO*. Dutch Research Council (NWO). https://www.nwo.nl/en/researchprogrammes/corona-fast-track-data

Early Childhood Development Agency. (2012, August 13). *Ensuring quality early childhood education and childcare services*. https://www.ecda.gov.sg/PressReleases/Pages/Ensuring-quality-early-childhood-education-and-childcare-services.aspx

Food and Nutrition Service, U.S Department of Agriculture. (2020). *FNS Responds to COVID-19 | USDA-FNS*. https://www.fns.usda.gov/coronavirus

Government of Alberta. (2020). *How child intervention works: Changes due to COVID-19*. https://www.alberta.ca/how-child-intervention-works.aspx

Government of Michigan. (2020a). *Answering your questions on pregnancy & COVID-19*. https://www.michigan.gov/documents/coronavirus/Pregnancy_Guide_691446_7.pdf

Government of Michigan. (2020b). *Caring for your new baby during COVID-19*. michigan.gov/documents/mdhhs/Newborn_Guide_1_691460_7.pdf

Government of Netherlands. (2020, April 25). *Start landelijke campagne tegen huiselijk geweld in coronacrisis | Nieuwsbericht | Rijksoverheid.nl*. https://www.rijksoverheid.nl/actueel/nieuws/2020/04/25/start-landelijke-campagne-tegen-huiselijk-geweld-in-coronacrisis

Government of Ontario. (2020a, April 2). *Ontario Strengthening Victim Services in Response to COVID-19 | Ontario Newsroom*. https://news.ontario.ca/en/release/56545/ontario-strengthening-victims-services-in-response-to-covid-19

Government of Ontario, M. of C. and Y. S. (2020b). *About Ontario’s Children Aid Societies*. Government of Ontario, Ministry of Children and Youth Services, Communications and Marketing Branch. http://www.children.gov.on.ca/htdocs/English/professionals/childwelfare/societies/index.aspx

Government of Quebec. (2020a). *Pregnancy, delivery and the postnatal period during the coronavirus disease (COVID-19) pandemic*. https://www.quebec.ca/en/health/health-issues/a-z/2019-coronavirus/information-for-pregnant-women-coronavirus-covid-19/

Government of Quebec. (2020b). *Québec Immunization Program*. https://www.quebec.ca/en/health/advice-and-prevention/vaccination/quebec-immunisation-program/

Government of Quebec. (2020c). *Questions and answers on education and families during the COVID-19 pandemic*. https://www.quebec.ca/en/health/health-issues/a-z/2019-coronavirus/answers-questions-coronavirus-covid19/questions-answers-education-families-covid-19/

Government of United Kingdom. (2020). *Coronavirus (COVID-19): Guidance for children’s social care services*. GOV.UK. https://www.gov.uk/government/publications/coronavirus-covid-19-guidance-for-childrens-social-care-services/coronavirus-covid-19-guidance-for-local-authorities-on-childrens-social-care

Health Link BC. (2020a). *B.C. Immunization Schedules*. HealthLink BC. https://www.healthlinkbc.ca/tools-videos/bc-immunization-schedules

Health Link BC. (2020b, July 24). *Routine Checkups*. HealthLink BC. https://www.healthlinkbc.ca/health-topics/ue5162

Health Promotion Board, Government of Singapore. (2020). *National Childhood Immunisation Schedule*. https://www.nir.hpb.gov.sg/nirp/eservices/immunisationSchedule

HM Treasury, UK Government. (2020, March 11). *Budget 2020*. https://www.gov.uk/government/publications/budget-2020-documents/budget-2020

Home Office, UK Government. (2020, August 17). *Domestic Abuse Bill 2020: Overarching factsheet*. GOV.UK. https://www.gov.uk/government/publications/domestic-abuse-bill-2020-factsheets/domestic-abuse-bill-2020-overarching-factsheet

Jeugdzord Nederland. (2020, July 2). *Beslisboom corona in jeugdzorg*. Jeugdzorg werkt. https://www.jeugdzorg-werkt.nl/gezond-en-veilig-werken/jeugdzorg-tijden-van-corona/over-de-beslisboom

Jong JGZ. (2020). *Coronavirus en jouw afspraak bij Jong JGZ | Jong JGZ*. https://www.jongjgz.nl/coronavirus

Key worker: Official list of UK personnel who can still send children to school. (2020, March 20). *The Guardian*. http://www.theguardian.com/world/2020/mar/20/key-worker-official-list-of-uk-personnel-who-can-still-send-children-to-school

Li, T. W. (2020, February 20). *Income inequality in Singapore falls to lowest level in almost two decades as household incomes rise* [Text]. The Straits Times. https://www.straitstimes.com/singapore/income-inequality-in-singapore-falls-to-lowest-levels-in-almost-two-decades

Lisa Johnson. (2020, April 2). COVID-19: Alberta expands eligibility for child care to include all essential workers | Edmonton Journal. *Edmonton Journal*. https://edmontonjournal.com/news/politics/covid-19-alberta-expands-eligibility-for-child-care-to-include-all-essential-workers

Marr, C., Hingtgen, S., Sherman, A., Windham, K., & Cox, K. (2020, May 8). *Temporarily Expanding Child Tax Credit and Earned Income Tax Credit Would Deliver Effective Stimulus, Help Avert Poverty Spike*. Center on Budget and Policy Priorities. https://www.cbpp.org/research/federal-tax/temporarily-expanding-child-tax-credit-and-earned-income-tax-credit-would

Medina, A. F. (2020, April 1). Singapore Announces Second COVID-19 Stimulus Package: Salient Features. *ASEAN Business News*. https://www.aseanbriefing.com/news/singapore-announces-second-covid-19-stimulus-package-salient-features/

Michigan Department of Health and Human Services. (2020). *MDHHS - COVID-19 and Immunizations*. https://www.michigan.gov/mdhhs/0,5885,7-339-73971_4911_4914-530653--,00.html

Ministry of Child and Family Development, Government of British Columbia. (2021, January 14). *Ministry of Children & Family Development Response to COVID-19*. Province of British Columbia. https://www2.gov.bc.ca/gov/content/family-social-supports/covid-19-information

Ministry of Children and Family Development, Government of British Columbia. (2020, April 8). *Province provides emergency fund for children with special needs | BC Gov News*. https://news.gov.bc.ca/releases/2020CFD0043-000650

Ministry of Education, Government of Ontario. (2020, April 5). *Archived—Get support for families*. Ontario. https://www.ontario.ca/page/get-support-families

Ministry of Health, Government of Ontario. (2020). *COVID-19 Guidance: Emergency Childcare Centres*. http://www.health.gov.on.ca/en/pro/programs/publichealth/coronavirus/docs/2019_child_care_guidance.pdf

Ministry of Health, Welfare and Sport, Government of the Netherlands. (2020, March 20). *COVID-19: Childcare for children of people working in crucial sectors*. https://www.government.nl/documents/publications/2020/03/20/childcare-for-children-of-people-working-in-crucial-sectors

Ministry of Social and Family Development, Government of Singapore. (2020a, April 6). *Impact of COVID-19 on Singaporeans and Supporting Measures*. https://www.msf.gov.sg/media-room/Pages/Impact-of-COVID-19-on-Singaporeans-and-Supporting-Measures.aspx

Ministry of Social and Family Development, Government of Singapore. (2020b, December 31). *Support For Singaporeans Affected by COVID-19*. https://www.msf.gov.sg/assistance/Pages/covid19relief.aspx

Murphy, K. (2020, March 28). *Australian government pumps $1bn into health and family violence services as coronavirus spreads*. The Guardian. http://www.theguardian.com/australia-news/2020/mar/29/australian-government-to-pump-1bn-into-health-and-family-violence-services-as-coronavirus-spreads

National Association of State Mental Health Program Directors. (2020). *National Association of State Mental Health Program Directors Weekly Update*. https://nasmhpd.org/sites/default/files/October_2_2020_NASMHPD_Weekly_Update.pdf

National Network to End Domestive Violence. (2020, March 27). NNEDV Welcomes Passage of the CARES Act, Additional Relief Still Needed for Survivors of Domestic and Sexual Violence. *NNEDV*. https://nnedv.org/latest_update/nnedv-welcomes-passage-cares-act-additional-relief-still-needed-survivors-domestic-sexual-violence/

Nederlandese Vereniging Voor Obstetrie & Gynaecologie. (2020). Perinatologische zorg in tijden van COVID-19. *NVOG*. https://www.nvog.nl/actueel/perinatologische-zorg-in-tijden-van-covid-19/

NSW Health. (2020a, August 9). *Immunisation services during COVID-19*. https://www.health.nsw.gov.au/immunisation/Pages/vaccination-advice-during-covid-19.aspx

NSW Health. (2020b, November 29). *Guidance for child and family health services*. https://www.health.nsw.gov.au/Infectious/covid-19/communities-of-practice/Pages/child-and-family.aspx

OECD. (2006). *Starting Strong II: Early Childhood Education and Care*. OECD. https://doi.org/10.1787/9789264035461-en

OECD. (2019). *OECD Family Database—OECD*. OECD Family Database. https://www.oecd.org/els/family/database.htm

OECD. (2020). *Income Distribution Database*. Income Distribution Database. https://stats.oecd.org/Index.aspx?DataSetCode=IDD

Ontario Public Health Association. (2020, October 30). *Your COVID-18 Summary for Oct. 30th—National Projections* [Personal communication].

Prime Minister of Australia. (2020, April 2). *Early childhood education and care relief package*. https://www.pm.gov.au/media/early-childhood-education-and-care-relief-package

Provincial Health Services Authority. (2020). *Antenatal visits during COVID-19 pandemic*. http://www.bccdc.ca/Health-Professionals-Site/Documents/COVID19_AntenatalVisitsDuringPandemic.pdf.

Ritchie, H., Ortiz-Ospina, E., Beltekian, D., Mathieu, E., Hasell, J., Macdonald, B., Giattino, C., & Roser, M. (2021a). *Coronavirus (COVID-19) Cases—Statistics and Research*. Our World in Data. https://ourworldindata.org/covid-cases

Ritchie, H., Ortiz-Ospina, E., Beltekian, D., Mathieu, E., Hasell, J., Macdonald, B., Giattino, C., & Roser, M. (2021b). *Coronavirus (COVID-19) Testing—Statistics and Research*. Our World in Data. https://ourworldindata.org/coronavirus-testing

Ritchie, H., Ortiz-Ospina, E., Beltekian, D., Mathieu, E., Hasell, J., Macdonald, B., Giattino, C., & Roser, M. (2021c). *Policy Responses to the Coronavirus Pandemic—Statistics and Research*. Our World in Data. https://ourworldindata.org/policy-responses-covid

Royal College of Obstetricians & Gynaecologists. (2020). *Coronavirus (COVID-19) Infection in Pregnancy—Version 12*. https://www.rcog.org.uk/globalassets/documents/guidelines/2020-10-14-coronavirus-covid-19-infection-in-pregnancy-v12.pdf

Santhanam, L. (2020). *Postnatal Maternal and Infant Care during the COVID-19 Pandemic: A guide for General Practice (Version 3)*. Royal College of General Practitioners. https://elearning.rcgp.org.uk/pluginfile.php/148864/mod_page/content/86/Postnatal%20Maternal%20and%20Infant%20Care%20during%20the%20COVID-19%20Pandemic%20-%20A%20guide%20for%20General%20Practice%20Version%203%20%2811.11.2020%29.pdf

Staniscuaski, F., Reichert, F., Werneck, F. P., de Oliveira, L., Mello-Carpes, P. B., Soletti, R. C., Almeida, C. I., Zandona, E., Ricachenevsky, F. K., Neumann, A., Schwartz, I. V. D., Tamajusuku, A. S. K., Seixas, A., Kmetzsch, L., & Parent in Science Movement†. (2020). Impact of COVID-19 on academic mothers. *Science*, *368*(6492), 724.1-724. https://doi.org/10.1126/science.abc2740

The Office of Governor Gretchen Whitmer, Government of Michigan. (2020, April 15). *Whitmer—Executive Order 2020-51: Expanding child care access during the COVID-19 pandemic—RESCINDED*. https://www.michigan.gov/whitmer/0,9309,7-387-90499_90705-526011--,00.html

The Royal Australian and New Zealand College of Obstetricians and Gynaecologists. (2020, August 6). *Info for Pregnant Women*. https://ranzcog.edu.au/statements-guidelines/covid-19-statement/information-for-pregnant-women

Vanderslott, S., Dadonaite, B., & Roser, M. (2013, May 10). *Vaccination*. Our World in Data. https://ourworldindata.org/vaccination

Werkgelegenheid, M. van S. Z. en. (2020, March 24). *Noodsteun om voedselbanken draaiende te houden—Nieuwsbericht—Rijksoverheid.nl* [Nieuwsbericht]. Ministerie van Algemene Zaken. https://www.rijksoverheid.nl/actueel/nieuws/2020/03/24/noodsteun-om-voedselbanken-draaiende-te-houden

Working Families. (2020). *Coronavirus (COVID-19) – What financial support is there for working families?* Working Families. https://workingfamilies.org.uk/articles/coronavirus-support/

World Bank. (2022). *World Bank Open Data | Data*. Open Data. https://data.worldbank.org/
